# Fujifilm SILVAMP TB-LAM for the Diagnosis of Tuberculosis in Nigerian Adults

**DOI:** 10.3390/jcm10112514

**Published:** 2021-06-06

**Authors:** Patricia Comella-del-Barrio, John S. Bimba, Ramota Adelakun, Konstantina Kontogianni, Bárbara Molina-Moya, Okoedoh Osazuwa, Jacob Creswell, Luis E. Cuevas, José Domínguez

**Affiliations:** 1 Institut d’Investigació Germans Trias i Pujol, CIBER Enfermedades Respiratorias (CIBERES), Universitat Autònoma de Barcelona, Carretera del Canyet, Camí de les Escoles s/n, Badalona, 08916 Barcelona, Spain; patricia.comella@e-campus.uab.cat (P.C.-d.-B.); bmolina@igtp.cat (B.M.-M.); 2 Zankli Research Centre and Department of Community Medicine, Bingham University, Karu 961105, Nigeria; bimbajs@yahoo.com (J.S.B.); okoedohosazuwa@gmail.com (O.O.); Luis.Cuevas@lstmed.ac.uk (L.E.C.); 3 Department of Clinical Sciences, Liverpool School of Tropical Medicine, Pembroke Place, Liverpool L3 5QA, UK; ramota.adelakun.19@ucl.ac.uk (R.A.); Nadia.Kontogianni@lstmed.ac.uk (K.K.); 4 Stop TB Partnership, TB REACH, 1218 Geneva, Switzerland; jacobc@stoptb.org

**Keywords:** tuberculosis, diagnosis, lipoarabinomannan, LAM, HIV, urine, point-of-care

## Abstract

There is a need for diagnostics for tuberculosis (TB) that are easy to use, able to screen non-sputum samples, and able to provide rapid results for the management of both immunocompromised and immunocompetent individuals. The Fujifilm SILVAMP TB LAM (FujiLAM) assay, a new non-sputum based point of need test for the diagnosis of TB, could potentially address most of these needs. We evaluated the performance of FujiLAM in HIV positive and HIV negative patients with presumptive TB attending three district hospitals in Nigeria. Consecutive patients were asked to provide urine samples on the spot, which were tested with FujiLAM. The results were compared against a positive culture and/or Xpert MTB/RIF as the reference standard. Forty-five patients had bacteriologically confirmed TB, and 159 had negative culture and Xpert MTB/RIF (no TB). The FujiLAM test was positive in 23 (sensitivity 65.7%, 95% CI = 48–80) HIV negative and seven (70%, 95% CI = 35–92) HIV positive patients with bacteriological confirmation of TB. FujiLAM was negative in 97 (specificity 99.0%, 95% CI = 94–100) HIV negative and 56 (93.3%, 95% CI = 83–98) HIV positive patients without TB. The FujiLAM test has good diagnostic accuracy for considering its application in both HIV positive and HIV negative patients with TB.

## 1. Introduction

Tuberculosis (TB) continues to cause high morbidity and mortality worldwide [1]. Despite increases in TB notifications in recent decades [2] and a major expansion in the use of the World Health Organization (WHO)-recommended molecular diagnostics (WRDs), 2.9 of the ten million estimated people who develop TB are missed by national TB programs each year.

A major drawback of current WRDs is the poor timeliness of test results, which often return to the clinic several hours or days later, when clinical decisions have been taken and the patients have left the premises [3]. It is recognized that, to be impactful, diagnostic test results need to be available at the time of patient management, to guide treatment initiation, and to reduce pre-treatment losses to follow-up [4]. Ideally, assays should be conducted at the point of need, using minimal laboratory skills and examining non- or minimally invasive clinical samples [5,6]. Moreover, large proportions of presumptive individuals cannot expectorate sputum or provide a high-quality sample, and therefore, non-sputum based tests could be helpful for many populations.

Current non-sputum based, point-of-need prototypes target serological markers, bacterial components, or detritus, including the lipoglycan and virulence factor lipoarabinomannan (LAM). LAM is a heat stable component of the outer cell wall of the bacilli that is released from metabolically active or degenerating bacteria of the genus Mycobacterium. LAM is filtered by the kidney and can be detected in urine, with test prototypes mentioned in the literature since the 1930s [7,8]. Although current LAM assays have low sensitivity, performing better in individuals with HIV and advanced immunosuppression [9], a recent prototype, Fujifilm SILVAMP TB LAM (FujiLAM, Fujifilm, Tokyo, Japan), is reported to have higher sensitivity in both HIV-infected and uninfected individuals [10,11], thanks to the use of high affinity monoclonal antibodies against *Mycobacterium tuberculosis*-specific LAM epitopes and a silver amplification step that increases the visibility of the test lines [11].

We report here a cross-sectional study to assess the diagnostic performance of FujiLAM in consecutive adults with presumptive TB attending ambulatory clinics in Nigeria.

## 2. Materials and Methods

This was a retrospective study of adults with signs and symptoms suggestive of TB attending TB diagnostic clinics at district hospitals of Abuja, Nigeria. Adults above 18 years old with presumptive TB [12] were enrolled consecutively at the time of submitting samples for diagnosis, regardless of their HIV status. Adults who had formerly been diagnosed as having TB or who had received TB treatment in the previous year were excluded.

**Figure 1:**
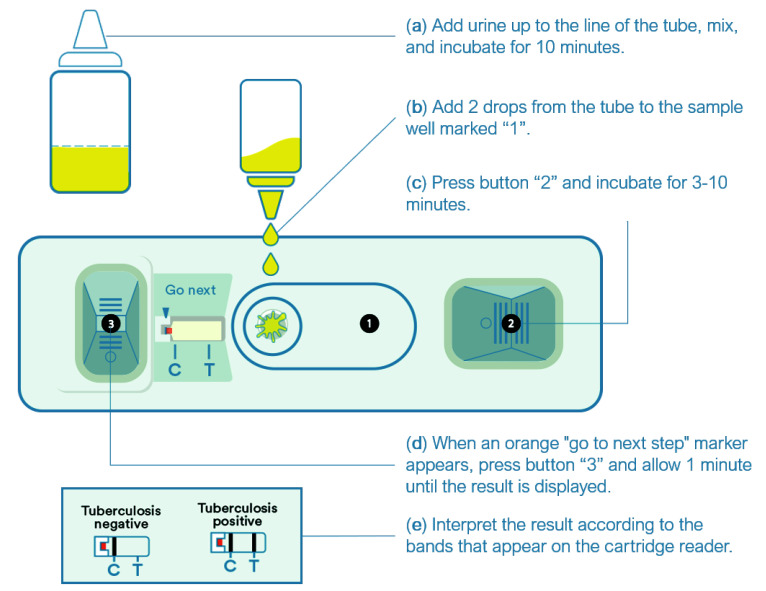
Outline of the procedures to perform the Fujifilm SILVAMP TB-LAM assay.

After obtaining written informed consent, participants were interviewed to obtain clinical and demographic information. Patients were asked to provide sputum, blood samples, and one midstream urine sample on-site for routine and study assays. All samples except urine were processed locally and were used for patient management. Sputum samples were tested with Xpert MTB/RIF (Cepheid, Sunnyvale, CA, USA), and cultured in solid media in duplicate using Löwenstein–Jensen medium. Urine samples were collected in sterile plastic containers and kept in cold boxes until processing for storage the same day. Samples were aliquoted (2 mL) into cryovials and transported frozen to the Institut d’Investigació Germans Trias i Pujol (Badalona, Spain) for LAM testing. One aliquot per participant was thawed at room temperature the day of testing, mixed with a vortex, and tested using FujiLAM following the manufacturers’ instructions [11]. Briefly, the reagent tube was filled with urine up to the indicator line, mixed without inverting, and incubated for 40 min at ambient temperature. During this incubation, the gold (Au)-conjugated primary antibody captured the 5-methylthio-D-xylofuranose-lipoarabinomannan antigen present in the patient’s urine. The tube was then mixed, and two drops of the contents of the tube were added to the sample well of the test cartridge. Immediately, we pressed the button 2 on the cartridge and waited 10 min until the orange mark appeared on the cartridge readout indicating “go to next step”. During this incubation, the sandwich immunocomplex was formed by binding to the immobilized secondary antibody. At the signal to proceed to the next step, the button 3 was pressed, releasing silver particles (10 um in diameter), which cluster around the gold particles and amplify the intensity of the cartridge reader band. The test results could be read approximately 1 min later on the test cartridge reader. Figure 1 shows schematically the test procedure. The test was considered positive if the control and test lines were visible (even if the line was faint) and negative if only the control line appeared. Tests without a control line were considered invalid and were repeated once. A video describing the test procedures is available at https://www.youtube.com/watch?v=aK-QtzkLBug (accessed 26 May 2021). The test lines were read by two investigators blinded to the patients’ condition and all other test results. In the case of disagreement with the result, the test was repeated once.

HIV status was assessed using two rapid antigen tests, and the viral loads of patients with HIV were assessed in plasma using the Xpert HIV-1 viral load (VL) assay (Cepheid, Sunnyvale, CA) according to the manufacturer’s instructions. The VL results were interpreted as detected, detected < 40 copies/mL, detected > 10^7^ copies/mL, undetected, and undetermined. The range of detection of the Xpert HIV-1 VL test was 40 to 10^7^ copies/mL (1.6 to 7.0 log_10_).

We used the chi-squared and Fisher’s exact tests to test parametric data and Student’s *t*-tests for continuous variables with normal distributions. Differences were considered statistically significant when the *p*-value was less than 0.05. Analysis was performed using SPSS (SPSS version 26.0, SPSS Inc, Chicago, IL, USA). Xpert and culture results were used to classify participants as bacteriologically confirmed if either the Xpert MTB/RIF or culture results were positive and as non-TB if both tests were negative. The sensitivity and specificity of the FujiLAM test were estimated using the combined results of Xpert MTB/RIF and culture as the reference standard (bacteriologically confirmed). We considered invalid FujiLAM results as negative, but annotated these results in separate rows. Written informed consent was obtained from all participants. The study was approved by the research ethics committees of the Liverpool School of Tropical Medicine, the Nigerian National Ethics Committee, and Ethics Committee of the Hospital Universitari Germans Trias i Pujol.

## 3. Results

Two hundred and four participants with a mean (SD) age of 37 (12.8) years were enrolled, as shown in Table 1. Thirty-seven (18.1%) had positive *M. tuberculosis* culture, 40 (19.6%) were Xpert MTB/RIF positive, and 45 (22.1%) culture or Xpert MTB/RIF positive (called bacteriologically confirmed). Four (10.8%) culture-positive participants were Xpert MTB/RIF negative, and three (7.5%) and four (10.0%) Xpert MTB/RIF-positive had negative or contaminated culture, respectively. One hundred and fifty-nine (77.9%) participants were culture and Xpert MTB/RIF negative (called not TB) (Table 1). Overall, 70 (34.3%) participants were HIV positive, 133 (65.2%) HIV negative and the HIV status was not known in one. Viral loads among HIV positive participants were undetectable in 11 (15.7%), <40 copies/mL in 16 (22.9%), and between 40 and 10^7^ copies/mL in 32 (45.7%), with three (4.3%) and eight (11.4%) participants having indeterminate and missing viral load results, respectively.

All 204 participants were tested with FujiLAM. Thirty-six (17.6%) were FujiLAM positive, 164 (80.4%) FujiLAM negative, and four (2.0%) had invalid results (Table 2). FujiLAM identified a similar proportion of bacteriologically confirmed individuals by HIV status, and was positive in 30 (66.7%) of 45 patients with bacteriologically confirmed TB, including 23 (65.7%) of the 35 HIV negative and seven (70%) of the ten HIV positive patients (*p* = 0.56). Among the 159 participants with no TB (culture and Xpert MTB/RIF negative), FujiLAM was positive in six (3.8%), invalid in four (2.5%), and negative in 149 (93.7%). Ninety-seven (99%) of the 98 HIV negative and 56 (93.3%) of the 60 HIV positive participants had negative/invalid FujiLAM results. The overall FujiLAM sensitivity and specificity were 66.7% (30/45) (95% CI = 51–80) and 96.2% (153/159) (95% CI = 92–98) among all participants, varying from 65.7% (95% CI = 48–80) and 99.0% (95% CI = 94–100) for HIV negative and 70.0% (95% CI = 35–92) and 93.3% (95% CI = 83–98) for HIV positive patients, respectively (Table 3). Positive and negative predictive values are also shown in Table 3 for patients with bacteriologically confirmed TB (Xpert or culture positive) and patients with no TB (Xpert and culture negative). The positive predictive value was higher among HIV negative patients (96%) than HIV positive (63%), and the negative predictive value was higher among HIV positive (94%) than HIV negative patients (89%). However, the differences were not statistically significant.

FujiLAM results had a similar pattern when analyzed using culture or Xpert MTB/RIF results singly, as shown in Table 2 and Table 3, or when disaggregated by gender. FujiLAM results by Xpert MTB/RIF grades and HIV status are shown in Table 2. Although the numbers were too small for statistical analysis, the proportion of participants with positive FujiLAM seemed to be higher among HIV negative participants with high Xpert grades (eight (73%) of 11) than among participants with very low Xpert/RIF grades (four (57%) of seven). However, this pattern was not observed among participants with HIV, as all six HIV positive participants were FujiLAM positive, independently of their Xpert MTB/RIF grade (Table 3). FujiLAM by HIV viral load is shown in Table 4 for bacteriologically confirmed, culture, or Xpert MTB/RIF positive participants and Xpert grade. Most of the patients had high HIV viral loads. The sensitivity seemed higher among patients with a high viral load, but the numbers were too small to conduct a statistical analysis. 

## 4. Discussion

We have evaluated the diagnostic accuracy of FujiLAM in urine samples of adults with signs and symptoms suggestive of TB attending three district hospitals in Abuja, Nigeria. We found a sensitivity and specificity of 65.7% and 99.0% among HIV negative patients and 70.0% and 93.3% among HIV positive patients. These results are higher than reported for the rapid LAM test AlereLAM, which has a reported sensitivity of 42% (95% CI = 31–55) among patients with HIV [13]. AlereLAM is recommended by the WHO for the complementary diagnosis of TB in patients with HIV, but not for HIV negative patients due to its low sensitivity in immune-competent individuals [13].

The overall sensitivity of FujiLAM in HIV positive patients was slightly higher than in HIV negative participants, but this difference was based on a small number of patients, and was not statistically significant. However, the higher sensitivity observed among immunosuppressed individuals with HIV viral loads between 40 and 10^7^ copies/mL (75.0%), was similar to studies of HIV positive patients in Ghana and South Africa [11,14]. The higher sensitivity of LAM among HIV positive individuals is said to reflect the increased concentration of LAM in urine due to the hematogenic spread of TB to the kidneys in immunosuppressed individuals [15]. However, LAM detection in urine is not limited to renal involvement [16], and is likely to be associated with the total bacterial burden of *M. tuberculosis* and the severity of disease [17].

The overall sensitivity among HIV negative patients (65.7%) met the minimum target of 65% for the WHO high-priority, non-sputum-based TB diagnostic tests [18]. Our results were higher than reported in a multicenter study of HIV negative patients in Peru and South Africa, which might be explained by the characteristics of the participants. FujiLAM is said to have higher sensitivity in patients with more advanced TB and higher *M. tuberculosis* loads in culture [19], and, as patients in Nigeria were recruited from district hospitals, a high proportion of our participants may have had advanced disease stages.

FujilAM specificity among HIV negative patients was very high (99%) and similar to other studies among HIV negative patients [18,19]. Specificity among HIV positive patients was marginally higher (93.3%) than reported by a study of bio-banked urine samples from HIV positive patients in three Sub-African countries [14], which reported a 90.8% specificity [11]. Patients with HIV experience multiple opportunistic infections, and positive FujiLAM results may well be false positives. However, it is also possible that these results reflect the difficulty of confirming the diagnosis in patients with disseminated TB disease. Culture and WRDs are imperfect tests, which have a lower performance in patients with HIV, extra-pulmonary, and disseminated TB, depending on the quality of the sample and whether the patient is excreting bacilli the day of sampling, and it is possible that patients who do not reach microbiological confirmation may have a missed TB diagnosis [20]. Our six patients with positive urine LAM results but negative sputum tests had the same clinical presentation as patients with bacteriologically confirmed TB. As it is likely that some patients with TB do not have bacilli in sputum in the absence of cavitation or when the disease is disseminated without communications with the airways (e.g., military TB), we cannot rule out that these patients may have been detected by a test based in urine that does not require expectoration of bacilli, and further studies are needed to confirm whether these were true or false positive results. However, it is also true that LAM is a cell wall compound that is present in most Mycobacteria species, and is not exclusive in *M. tuberculosis*. The FujiLAM assays combine high-affinity monoclonal antibodies directed towards the largely *M. tuberculosis*-specific MTX-LAM epitopes, which are expected to increase the sensitivity of the assay by using a silver-based amplification step without affecting the specificity. Despite the potential cross reactivity risk, we feel that the assay would be useful in the field, as it would allow the rapid identification of about two-thirds of patients with TB at the time of the first consultation. The assays would need to be incorporated into diagnostic algorithms, as patients would need to undergo further confirmatory tests to confirm the presence of MTB and then be screened for drug resistance.

Urine is a readily available, non-invasive sample, which would be especially useful in patients unable to expectorate, such as children, the elderly, and adults without a productive cough and individuals with disseminated, extra-pulmonary, and non-cavitary disease. Unfortunately, the information regarding whether the patients included in our study presented cavitary disease or not was not available with the data we collected. FujiLAM is simple to use, does not require sputum samples, which minimizes the risk of aerosols, does not require additional instrumentation, and can be used in decentralized laboratories [21]. Therefore, FujiLAM is a promising test for the early detection and treatment of TB in people with signs and symptoms suggestive of TB, with a particular relevance for low resource health centers in low- and middle-income countries with the highest burden of TB.

Our study has several limitations. We only included patients able to provide sputum, which may underestimate the potential of FujiLAM to identify patients who are difficult to diagnose. In addition, the number of bacteriologically confirmed TB cases is small, especially for patients with HIV, and we were unable to follow-up with participants, which could have re-classified some individuals with negative culture and Xpert MTB/RIF tests as positive, potentially increasing the specificity.

An important issue for the wider use of FujiLAM is that the test costs are currently too high for its wider implementation outside of a research setting. FujiLAM is likely to be most useful in locations with limited resources, and tiered pricing mechanisms will be needed to facilitate access to the tests according to need. Moreover, further cost effectiveness studies are needed. A study in South Africa and Malawi examining patients with HIV reported that FujiLAM combined with Xpert MTB/RIF was more cost effective than using Xpert MTB/RIF alone [22]. However, more studies are needed to assess its cost effectiveness in HIV negative patients, especially at lower levels of the health system in low resource settings.

Despite the development and scale-up of newer, more sensitive TB diagnostic tests over the last decade, these have not lived up to their early promise, because they remain too slow, expensive, and resource-intensive (liquid culture), have been mostly implemented centrally, or have been found to have high diagnostic accuracy only among a subset of patients (e.g., urine LAM for severe HIV-associated illness). However, there is an emerging pipeline of new TB tests and tools that could allow rapid, accurate, point-of-need diagnosis. These tests are probably insufficient when used individually, but their performance could be optimized when used in combination as novel diagnostic algorithms. This is the case of the new generation urine LAM rapid test that we have studied: the FUJILAM test. Although the test has limited sensitivity, it would be able to detect two thirds of patients with bacteriologically confirmed TB at the time of the first consultation. Future research, therefore, should evaluate the potential of FujiLAM in immunocompetent adults and children attending primary healthcare facilities and in patients with extrapulmonary and non-cavitary TB. Further studies are also needed to develop diagnostic algorithms that incorporate drug susceptibility testing for patients identified by FujiLAM at the lower levels of the healthcare system, and whether its combination with other screening tests, such as C Reactive Protein, could be used to develop point-of-care diagnostic algorithms.

There is also a clear need of new technology and stronger efforts in the development, validation, and market shaping initiatives to expand the use of these devices. FujiLAM manufacturing facilities are currently being expanded, and its manufacturer plans to apply for WHO endorsement in 2022. Other LAM prototype developers are also making strides to develop low-cost lateral flow assays with improved monoclonal antibodies, which are expected to become available as research use-only prototypes in 2022, while novel LAM concentration methods are being tested in the field with preliminarily good performance.

In conclusion, testing urine samples with FujiLAM in HIV positive and HIV negative patients with presumptive TB has a higher performance than current urine LAM assays. The use of the FujiLAM test would facilitate the detection and initiation of TB treatment on the same day of consultation in primary health centers.

## Figures and Tables

**Table 1:** Characteristics of the study participants.

		All (*n* = 204)	Not TB (*n* = 159)	Bact + TB (*n* = 45)	*p*-Value
Age	mean (SD)	37.0 (12.8)	37.65 (13.0)	34.60 (12.0)	0.160
Sex	Male	95 (46.6%)	69 (43.4%)	26 (57.8%)	0.088
	Female	109 (53.4%)	90 (56.6%)	19 (42.2%)	
HIV	Negative	133 (65.2%)	98 (61.6%)	35 (77.8%)	0.087
	Positive	70 (34.3%)	60 (37.7%)	10 (22.2%)	
	Unknown	1 (0.5%)	1 (0.6%)	0 (0.0%)	
Culture	Negative	155 (76.0%)	151 (95.0%) ^a^	4 (8.9%) ^a^	**<0.01**
	Positive	37 (18.1%)	0 (0.0%) ^a^	37 (82.2%) ^a^	
	Contaminated	12 (5.9%)	8 (5.0%)	4 (8.9%)	
Xpert	Negative	164 (80.4%)	159 (100%) ^a^	5 (11.1%) ^a^	**<0.01**
	Positive	40 (19.6%)	0 (0.0%) ^a^	40 (88.9%) ^a^	

SD: standard deviation. The *p*-value shows the significant differences observed for each variable between the proportions of bacteriologically confirmed TB patients and non-TB patients. ^a^ Column proportions that differ significantly with a *p*-value under 0.05 (in bold).

**Table 2:** FujiLAM test results by TB and HIV status.

	All			HIV Negative		HIV Positive		
FujiLAM	Negative *n* (%)	Positive *n* (%)	Invalid *n* (%)	Negative *n* (%)	Positive *n* (%)	Negative *n* (%)	Positive *n* (%)	Invalid *n* (%)
Bact + TB ^a^	15 (33.3%)	30 (66.7%)	0 (0.0%)	12 (34.3%)	23 (65.7%)	3 (30.0%)	7 (70.0%)	0 (0.0%)
(B +) male	9 (34.6%)	17 (65.4%)	0 (0.0%)	7 (35.0%)	13 (65.0%)	2 (33.3%)	4 (66.7%)	0 (0.0%)
(B +) female	6 (31.6%)	13 (68.4%)	0 (0.0%)	5 (33.3%)	10 (66.7%)	1 (25.0%)	3 (75.0%)	0 (0.0%)
Culture pos	11 (29.7%)	26 (70.3%)	0 (0.0%)	9 (30.0%)	21 (70.0%)	2 (28.6%)	5 (71.4%)	0 (0.0%)
Xpert pos	11 (27.5%)	29 (72.5%)	0 (0.0%)	11 (32.4%)	23 (67.6%)	0 (%)	6 (100%)	0 (0.0%)
High	3 (23.1%)	10 (76.9%)	0 (0.0%)	3 (27.3%)	8 (72.7%)	0 (%)	2 (100%)	0 (0.0%)
Medium	5 (29.4%)	12 (70.6%)	0 (0.0%)	5 (35.7%)	9 (64.3%)	0 (%)	3 (100%)	0 (0.0%)
Low	0 (%)	3 (100%)	0 (0.0%)	0 (0.0%)	2 (100%)	0 (%)	1 (100%)	0 (0.0%)
Very Low	3 (42.9%)	4 (57.1%)	0 (0.0%)	3 (42.9%)	4 (57.1%)	0 (%)	0 (0.0%)	0 (0.0%)
Culture neg	143 (92.2%)	8 (5.2%)	4 (2.6%)	92 (97.9%)	2 (2.1%)	51 (85.0%)	5 (8.3%)	4 (6.7%)
Culture cont	10 (83.3%)	2 (16.7%)	0 (0.0%)	8 (88.9%)	1 (11.1%)	2 (66.7%)	1 (33.3%)	0 (0.0%)
Xpert neg	153 (93.3%)	7 (4.3%)	4 (2.4%)	98 (99.0%)	1 (1.0%)	55 (85.9%)	5 (7.8%)	4 (6.3%)
Not TB ^b^	149 (93.7%)	6 (3.8%)	4 (2.5%)	97 (99.0%)	1 (1.0%)	52 (86.7%)	4 (6.7%)	4 (6.7%)
Total	164 (80.4%)	36 (17.6%)	4 (2.0%)	109 (82.0%)	24 (18.0%)	55 (78.6%)	11 (15.7%)	4 (5.7%)

^a^ Positive culture and/or Xpert. ^b^ Negative culture and Xpert.

**Table 3:** Sensitivity and specificity of FujiLAM test by HIV status.

	All		HIV Negative		HIV Positive	
	**Sensitivity** **[*n*/N, %,** **95% CI]**	**Specificity** **[*n*/N, %,** **95% CI]**	**Sensitivity** **[*n*/N, %,** **95% CI]**	**Specificity** **[*n*/N, %,** **95% CI]**	**Sensitivity** **[*n*/N, %,** **95% CI]**	**Specificity** **[*n*/N, %,** **95% CI]**
Bact + TB ^a^	30/45, 66.7%, 51–80	153/159, 96.2%, 92–98	23/35, 65.7%, 48–80	97/98, 99.0%, 94–100	7/10, 70.0%, 35–92	56/60, 93.3%, 83–98
Culture	26/37, 70.3%, 53–84	147/155, 94.8%, 90–98	21/30, 70.0%, 50–85	92/94, 97.9%, 92–100	5/7, 71%, 31–95	55/60, 91.7%, 81–97
Xpert	29/40, 72.5%, 56–85	157/164, 95.7%, 91–98	23/34, 67.6%, 49–82	98/99, 99.0%, 94–100	6/6, 100%, 52–100	59/64, 92.2%, 82–97
	**PPV** **[*n*/N, %,** **95% CI]**	**NPV** **[*n*/N, %,** **95% CI]**	**PPV** **[*n*/N, %,** **95% CI]**	**NPV** **[*n*/N, %,** **95% CI]**	**PPV** **[*n*/N, %,** **95% CI]**	**NPV** **[*n*/N, %,** **95% CI]**
Bact + TB ^a^	30/36, 83%, 67–94	153/173, 88%, 83–93	23/24, 96%, 79–99	97/109, 89%, 82–94	7/11, 63%, 31–89	56/59, 94%, 86–99

^a^ Positive culture and/or Xpert. Specificity estimated considering not TB cases as not having TB. CI: confidence interval. PPV: Positive predictive value. NPP: Negative predictive value.

**Table 4:** Positive FujiLAM test results among HIV positive participants by TB status and HIV RNA viral load.

	HIV Viral Load				
	Undetected	<40 cps/mL	40–10^7^ cps/mL	Indeterminate	Missing
Bact + TB ^a^	1/1 (100%)	1/2 (50%)	3/4 (75%)	0/0 (0%)	2/3 (67%)
Culture pos	0/0 (0%)	1/2 (50%)	3/3 (100%)	0/0 (0%)	1/2 (50%)
Xpert pos	1/1 (100%)	1/1 (100%)	2/2 (100%)	0/0 (0%)	2/2 (100%)
High	0/0 (0%)	0/0 (0%)	1/1 (100%)	0/0 (0%)	1/1 (100%)
Medium	1/1 (100%)	0/0 (0%)	1/1 (100%)	0/0 (0%)	1/1 (100%)
Low	0/0 (0%)	1/1 (100%)	0/0 (0%)	0/0 (0%)	0/0 (0%)
Very Low	-	-	-	-	-
Culture neg	1/10 (10%)	0/14 (0%)	4/28 (14%)	0/3 (0%)	0/5 (0%)
Culture contaminated	0/1 (0%)	0/0 (0%)	0/1 (0%)	0/0 (0%)	1/1 (100%)
Xpert neg	0/10 (0%)	0/15 (0%)	5/30 (17%)	0/3 (0%)	0/6 (0%)
Not TB ^b^	0/10 (0%)	0/14 (0%)	4/28 (14%)	0/3 (0%)	0/5 (0%)
Bact + TB					
Sensitivity	100%, 0.05–1	50%, 0.03–1	75%, 0.2–1	0%	67%, 0.1–1
Not TB					
Specificity	100%, 0.7–1	100%, 0.7–1	86%, 0.7–1	100%, 0.3–1	100%, 0.5–1

^a^ Positive culture and/or Xpert. ^b^ Negative culture and Xpert.

## Data Availability

The data presented in this study are available on request from the corresponding author. The data are not publicly available due to privacy reasons.
